# Efficacy of four vaginal progesterones for luteal phase support in frozen‐thawed embryo transfer cycles: A randomized clinical trial

**DOI:** 10.1002/rmb2.12300

**Published:** 2019-09-16

**Authors:** Reiko Shiba, Masayuki Kinutani, Shinichiro Okano, Reo Kawano, Yuko Kikkawa

**Affiliations:** ^1^ Kinutani Women’s Clinic Hiroshima Japan; ^2^ Center for Integrated Medical Research Hiroshima University Hospital Hiroshima Japan; ^3^ Present address: Katsuki Ladies Clinic Hiroshima Japan

**Keywords:** clinical pregnancy, frozen‐thawed embryo transfer, luteal phase support, randomized clinical trial, vaginal progesterone

## Abstract

**Purpose:**

To investigate the efficacy of four vaginal progesterones, Lutinus, Utrogestan, Luteum, and Crinone, as luteal phase support (LPS) in frozen‐thawed embryo transfer (Frozen‐ET) cycles.

**Methods:**

Patients undergoing autologous Frozen‐ET of one cleavage‐stage embryo or one blastocyst. Two hundred fifty‐nine Frozen‐ET cycles were randomized to four groups for LPS: Lutinus, Utrogestan, Luteum, and Crinone. The clinical pregnancy rate (CPR), fetal heartbeat rate (FHR), and miscarriage rate (MR) were analyzed using the Mann‐Whitney or Kruskal‐Wallis test and Fisher exact test.

**Results:**

Two hundred thirty‐five Frozen‐ET cycles were analyzed: 63 cycles in the Lutinus group, 60 in the Utrogestan group, 56 in the Luteum group, and 56 in the Crinone group. No significant differences were observed between the four groups in CPR (Lutinus, Utrogestan, Luteum, and Crinone: 34.9%, 33.3%, 37.5%, and 35.7%, respectively; *P* = .976), FHR (26.9%, 31.6%, 30.3%, and 25.0%, respectively; *P* = .857), and MR (31.8%, 10.0%, 19.0%, and 30.0%, respectively; *P* = .306). Multivariate logistic regression analysis also revealed that there were no statistically significant differences between the four groups with regard to CPR, FHR, and MR.

**Conclusion:**

There was no clinically significant difference in pregnancy outcomes between the four vaginal progesterone groups for LPS in Frozen‐ET cycles.

## 1 INTRODUCTION

Currently, an increasing number of assisted reproductive treatments (ART) are being performed worldwide,^1^ and in particular, the use of frozen‐thawed embryo transfers (Frozen‐ET) is increasing, as this technique is commonly used as an alternative to fresh embryo transfer (Fresh‐ET).^1^ One of the reasons for the increase in the number of Frozen‐ET procedures is the improvement of the technology used to freeze embryos.^2^, ^3^ The Frozen‐ET technique was developed so that embryos left over after being obtained for in vitro fertilization (IVF) and after Fresh‐ET is completed can be thawed later and transferred in accordance with the mother's menstrual cycle for the purpose of another pregnancy. In addition, freezing embryos and storing them eliminates the need for the patient to undergo repeated egg retrieval procedures; therefore, it allows the endometrium to maintain the normal hormonal environment, which increases the pregnancy rate.^4^, ^5^ The use of the Frozen‐ET technique also avoids the risk of ovarian hyperstimulation syndrome.

It is a well‐known fact that luteal phase support (LPS) in ART is essential for the implantation and maintenance of pregnancy.^6^ However, in contrast to Fresh‐ET, the absence of endogenous serum progesterone (P4) secretion before frozen embryo transfer in hormone replacement therapy cycles (HRT‐FET) results in the need for exogenous P4 formulations at approximately pregnancy weeks 8 to 10.^6^, ^7^, ^8^ Thus, it is important to identify the dose of vaginal preparation alone that will facilitate continuation of the pregnancy.

There are two Frozen‐ET methods. Frozen‐ET that utilizes the patient's natural ovulation cycle does not involve the use of hormone administration, which reduces the burden placed on the patient. However, as the day of ovulation must be identified, this method requires that the patient be examined frequently, and it does not allow freedom in selecting the day on which the transfer is to take place. On the other hand, HRT‐FET, ovulation, and corpus luteum formation typically do not occur, and endometrial preparation requires exogenous P4 replacement. Since this method requires that the endometrium be artificially prepared, it gives patients the freedom to choose the date the transfer is to take place and keeps patient hospital visits to a minimum. Although there have been meta‐analysis studies done on both natural ovulation cycle with Frozen‐ET and HRT‐FET, they did not find significant differences in terms of the clinical pregnancy rate (CPR), ongoing pregnancy rate (OPR), or delivery rate.^9^ As a result, HRT‐FET seems to be preferable among working women.

Methods for administering P4 formulations in HRT‐FET are divided into intramuscular, oral, and vaginal administrations. Oral preparations, however, are subject to liver metabolism and are avoided owing to poor bioavailability and inferior pregnancy outcomes.^10^ Intramuscular injection is highly effective, but self‐administration is difficult and intense pain occurs at the site of injection. The long‐term use of intramuscular injection poses a serious burden, so patient satisfaction is higher with vaginal preparations.^11^ Many studies have shown the effect of vaginal supplements in Fresh‐ET cycles, in which vaginal P4 preparations and intramuscular injections have equivalent pregnancy rates.^12^, ^13^, ^14^ For these reasons, many medical facilities have shifted from intramuscular injections to vaginal preparations. In fact, according to a 2012 online survey, the usage rate of vaginal preparation as monotherapy is 77%, making it the most popular method.^15^

Vaginal P4 has been used for a long time in many countries, and many reports have shown its effectiveness in Fresh‐ET.^12^, ^14^, ^16^, ^17^, ^18^, ^19^, ^20^, ^21^ Although vaginal preparations are effective when used in Fresh‐ET according to a systematic review in 2018,^22^ a review in 2014 found that the data for their use in HRT‐FET were insufficient and the timing of LPS, method of application, and dose were all unknown.^6^ These are serious issues given that the number of Frozen‐ET procedures performed worldwide is increasing.

In Japan, unlike overseas, oral, in‐hospital vaginal, or intramuscular injection P4 preparation has long been used for luteal support in ART. In 2014, the P4 vaginal agent Lutinus was first approved, and in 2016, Utrogestan, Luteum, and Crinone were released successively. We conducted a prospective, randomized controlled trial with HRT‐FET in 2018 using three vaginal P4 suppositories: Lutinus, Utrogestan, and Luteum, and found no significant difference between the three groups in the CPR, OPR, and miscarriage rate (MR).^23^ During that study, Crinone was released, so this time, we conducted a new prospective, randomized comparative study on the use of four vaginal P4 suppositories in HRT‐FET, Lutinus, Utrogestan, Luteum, and Crinone, in order to investigate CPR, fetal heartbeat rate (FHR), and MR.

## 2 MATERIALS AND METHODS

### 2.1 Study design

This prospective randomized, open‐label, exploratory, parallel‐group controlled study was performed in a private infertility clinic (Kinutani Women's Clinic, Hiroshima, Japan) from December 1, 2016, to December 30, 2017. The protocol was approved by the Ethics Committee of the Kinutani Women's Clinic (ethical review number 2016‐1110‐1), and this study was registered in the UMIN Clinical Trials Registry (registration number UMIN000032997).

### 2.2 Study population

As this study was an exploratory study, no particular sample size was established. We excluded patients who had contraindications that were described on the drug package. This was the only exclusion criterion; we used no other restrictions such as age or cause of infertility. Thus, we requested the participation of all patients who visited our clinic on an outpatient basis, underwent egg retrieval, and had at least one embryo frozen using the vitrification method for cryopreservation. Patients who consented to study participation were enrolled in the study. The patients underwent autologous Frozen‐ET of one cleavage‐stage embryo or one blastocyst. Patients who desired to transfer two embryos or those who desired the use of a different preparation method were not permitted to participate in this study. Patients were permitted to participate two or more times.

Even if the patients participated in the study more than once, we allocated them to any of the four drug groups. Therefore, sometimes patients were assigned to the same vaginal drug used previously.

### 2.3 Randomization and intervention

All patients were given a registration number on the basis of the order of their referral. Then, a specific researcher generated a computer‐based random allocation table, and all patients were randomly assigned to one of the four study groups.

Those assigned to the first study group received 100 mg of a vaginal P4 tablet three times daily (Lutinus, Ferring Pharmaceuticals); those assigned to the second group received 200 mg of vaginal P4 capsules (Utrogestan, FUJIFILM Pharmaceuticals) three times daily; those assigned to the third group received a 400‐mg vaginal suppository (Luteum, ASKA Pharmaceutical) twice daily; and those in the fourth group received 90 mg of a vaginal gel (Crinone, Merckserono) once daily. The randomization lists were kept on a password‐protected computer.

### 2.4 Study protocol

All patients underwent an IVF cycle with an agonist, antagonist, and mild stimulant protocol. IVF, intracytoplasmic sperm injection (ICSI), or both methods were used in the process. Confirmation of fertilization was tested for at 16‐19 hours after IVF or ICSI. Several embryologists affiliated with the clinic performed cryopreservation of early embryos 2‐3 days after egg retrieval and cryopreservation of blastocysts 4‐7 days after egg retrieval. The vitrification method of cryopreservation was used in all cases. Before freezing, the early embryos were assessed using the Veek method,^24^ and the blastocysts were assessed using the Gardner method.^25^ In the case of several early embryos and blastocysts, the decision regarding whether to freeze them was left to four physicians affiliated with this clinic.

The HRT protocol was conducted using the gonadotropin‐releasing hormone agonist via nasal drops for down‐regulation 1‐2 weeks before the start of menstruation. We started with the use of estrogen (E2) tape (Estrana tape, Hisamitsu Pharmaceutical Co., Inc.) or E2 gel (L’estrogel 0.06%, FUJIFILM Pharmaceuticals), which is a transdermal E2 preparation, from menstrual cycle days 2‐5, gradually increased the dosage, checked whether the endometrial thickness was at least 7.0 mm on menstrual cycle days 12‐15, and then started P4 vaginal suppositories (day 0), with embryo transfer being performed on days 2 and 3 for a cleavage‐stage embryo and on day 5 for a blastocyst. In some cases, in which the endometrial thickness was <7.0 mm, the period of E2 administration was extended. It has been reported that when the endometrium is <7 mm, the pregnancy rate decreases.^26^ However, in some patients, the endometrium does not become thicker even if E2 is increased or prolonged. Thus, embryo transfer was not canceled because of insufficient thickness in any case. Single embryo transfer of a cleavage‐stage embryo or blastocyst was performed. If the patient had a positive beta‐human chorionic gonadotropin blood test result in pregnancy week 4, fetal growth was assessed weekly thereafter by transvaginal ultrasonography. The administration of vaginal suppositories was continued until pregnancy week 10 or pregnancy termination.

The primary outcome measure of this study was CPR, and the secondary outcome measures were FHR and MR.

### 2.5 Adverse events

All adverse reactions other than the side effects described on the drug package, such as thrombosis, headache, somnolence, genital bleeding, and diarrhea, were recorded. Patients were able to withdraw from the study if severe adverse events occurred.

### 2.6 Statistical analysis

Clinical pregnancy rate was assessed by checking for a gestational sac via transvaginal ultrasonography during pregnancy week 5. FHR was assessed during pregnancy week 7. MR was also assessed during pregnancy week 8. Pairwise comparisons between the four groups were performed using the Mann‐Whitney or Kruskal‐Wallis test for the continuous variables and the Fisher exact test for categorical variables. Univariable and multivariable logistic regression models were applied to investigate the effect of covariates (age, body mass index [BMI], and number of previous transfers) on CPR, FHR, and MR. All statistical analyses were performed with the statistical software SAS 9.4 (SAS Institute Inc). The level of significance for the univariate and multivariate analyses was set to <5% (*P* < .05).

## 3 RESULTS

We evaluated 259 cycles for eligibility, of which 254 were randomized to four study groups. Twenty‐four cycles could not be followed up or the patients discontinued the study. Reasons for dropping out of the study were as follows:

Per patients’ request, participation in five cycles was declined even after judging that there was no problem in the evaluation of eligibility. Two cycles were withdrawn from the Lutinus group because two patients wanted to transfer two embryos. Three cycles were withdrawn from the Utrogestan group because two patients wanted another drug, and one patient had no implantable embryo. Eight cycles were withdrawn from the Luteum group because one patient wanted another drug, four patients wanted to transfer two embryos, two patients had no implantable embryo, and one patient had severe vaginal bleeding and changed the drug. Six cycles were withdrawn from the Crinone group because three patients wanted to transfer two embryos, two patients had no implantable embryo, and one patient wanted another drug. No serious adverse events occurred in the women involved in this study.

**Figure 1:**
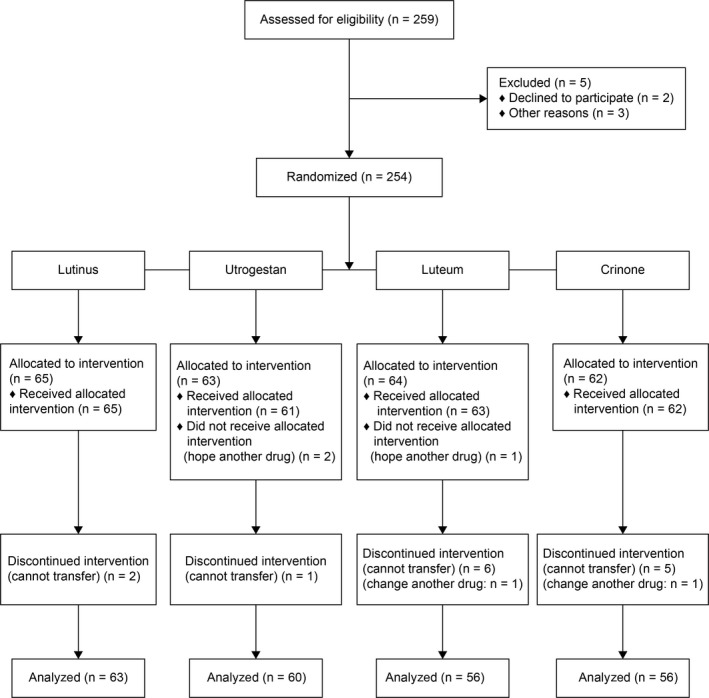
CONSORT flow diagram of the study. The diagram shows participant flow through the study, including patient eligibility, reasons for exclusion, treatment group allocation, loss to follow‐up, and number of cycles included in the final analysis

As a result, the number of cycles included in the final analysis was 235 (Figure 1). The number of patients who participated in this study was 183; thus, the average participation was 1.2 times. The number of cycles assigned to the same vaginal P4 as the previously assigned cycle was only 10.

The patient demographics are summarized in Table 1. Blastocysts that were assessed as 3BB or higher using the Gardner classification were identified as high‐quality embryos. The results showed no significant differences between the study groups regarding the baseline characteristics: age (*P* = .169), BMI (*P* = .384), pregnancy history (*P* = .732), previous transfers (*P* = .679), endometrial thickness (*P* = .132), transferred embryo (*P* = .909), and quality of the blastocyst (*P* = .384).

**Table 1:** Patient demographics for frozen embryo transfer cycles using four vaginal progesterones for luteal phase support

Group	Lutinus (n = 63)	Utrogestan (n = 60)	Luteum (n = 56)	Crinone (n = 56)	*P*‐value
Age (y)	37.0 (33.0‐40.0)	35.0 (32.0‐39.0)	37.5 (33.0‐41.0)	36.0 (33.0‐38.0)	.169
BMI (kg/m^2^)	20.8 (18.9‐22.2)	20.2 (18.7‐21.7)	20.2 (18.8‐21.8)	19.9 (18.5‐21.0)	.384
Pregnancy history					
Primary	30 (47.6)	31 (51.6)	26 (46.4)	23 (41.0)	.732
Secondary	33 (52.3)	29 (48.3)	30 (53.5)	33 (58.9)	
Previous transfers (times)					
0	21 (33.3)	24 (40.0)	15 (26.7)	15 (26.7)	.679
1	16 (25.4)	17 (28.3)	16 (28.5)	16 (28.5)	
≥2	26 (41.2)	19 (31.6)	25 (44.6)	25 (44.6)	
Endometrial thickness (mm)	11.2 (10.2‐12.8)	10.8 (9.9‐12.1)	10.5 (9.9‐11.7)	11.0 (9.75‐12.0)	.132
Transferred embryo					
Cleavage‐stage	31 (49.2)	30 (50.0)	25 (44.6)	25 (44.6)	.909
Blastocyst	32 (50.7)	30 (50.0)	31 (55.3)	31 (55.3)	
Quality of blastocyst					
High	23 (71.8)	25 (83.3)	21 (67.7)	20 (64.5)	.384
Poor	9 (28.1)	5 (16.6)	10 (32.2)	11 (35.4)	

Note

Data are presented as a median (IQR) or count (%). The IQR represents the 25th and 75th percentiles. The *P*‐value is >.05 for all variables when the four groups are compared.

IQR, interquartile range.

Although we consider the median age of the patients (35‐37 years) to be higher than that of those in other countries, this age group is commonly dealt with in fertility clinics located in Japan.

Pregnancy outcome data are shown in Table 2. There were no statistically significant differences between the four groups in the rates of CPR (Lutinus, Utrogestan, Luteum, and Crinone: 34.9%, 33.3%, 37.5%, and 35.7%, respectively; *P* = .976), FHR (26.9%, 31.6%, 30.3%, and 25.0%, respectively; *P* = .858), and MR (31.8%, 10.0%, 19.0%, and 30.0%, respectively; *P* = .306). The crude odds ratios (ORs) for these comparisons are shown in Table 3. We did not find any significant difference between the four study groups regarding CPR, FHR, and MR in univariate OR. We performed a multivariate logistic regression analysis to eliminate the confounding effects of age, BMI, and the number of previous transfers (Table 3). We also found that after controlling for confounders, there was no significant difference between the study groups.

**Table 2:** Comparison of pregnancy outcomes in 235 cycles of frozen‐thawed embryo transfer between four vaginal progesterones

	Lutinus (n = 63)	Utrogestan (n = 60)	Luteum (n = 56)	Crinone (n = 56)	*P*‐value
Clinical pregnancy rate	22 (34.9)	20 (33.3)	21 (37.5)	20 (35.7)	.976
Fetal heart beat	17 (26.9)	19 (31.6)	17 (30.3)	14 (25.0)	.858
Miscarriage rate	7 (31.8)	2 (10.0)	4 (19.0)	6 (30.0)	.306

Note

Data are presented as a count (%).

**Table 3:** Univariate and multivariate analyses of the clinical outcomes between the four vaginal progesterones

Outcome	Group	Univariate analysis		Multivariate analysis	
		OR (95% CI)	*P*‐value	OR (95% CI)	*P*‐value
Clinical pregnancy rate	Lutinus^a^	1		1	
	Utrogestan	0.93 (0.44‐1.97)	.853	0.42 (0.13‐1.30)	.131
	Luteum	1.12 (0.53‐2.37)	.770	0.60 (0.21‐1.75)	.351
	Crinone	1.04 (0.49‐2.20)	.927	0.76 (0.27‐2.18)	.611
Fetal heart beat	Lutinus^a^	1		1	
	Utrogestan	1.25 (0.58‐2.73)	.569	0.61 (0.19‐1.92)	.394
	Luteum	1.18 (0.53‐2.62)	.685	0.53 (0.17‐1.67)	.279
	Crinone	0.90 (0.40‐2.05)	.806	0.61 (0.20‐1.88)	.390
Miscarriage	Lutinus^a^	1		1	
	Utrogestan	0.24 (0.04‐1.32)	.101	0.17 (0.02‐1.83)	.146
	Luteum	0.50 (0.12‐2.07)	.342	0.55 (0.10‐3.14)	.503
	Crinone	0.92 (0.25‐3.41)	.899	0.82 (0.17‐3.92)	.807

Note

Data are presented as an OR with 95% CI.

CI, confidence interval; OR, odds ratio.

a The Lutinus group was considered as the reference group for comparison. Adjusted variables: age, body mass index, and number of previous transfers.

## 4 DISCUSSION

This study's results indicated that no significant differences were found between the four preparations in terms of CPR, FHR, and MR.

Currently, vaginal suppositories are commonly used throughout the world for LPS in ART.^15^ A systematic review conducted in 2018 concluded that there was no difference between four vaginal preparations when used in Fresh‐ET in terms of safety or effectiveness.^22^ However, an insufficient number of studies have compared the effectiveness of four preparations in HRT‐FET. Lan et al indicated that there was no difference between the Crinone group (90 mg/d) and Utrogestan group (200 mg three times per day) in terms of CPR in HRT‐FET.^27^ This result is consistent with our study's finding that no significant differences were found between the four vaginal formulations in terms of CPR. Other studies have shown the effectiveness of vaginal preparations in HRT‐FET 28, 29, 30, 31, 32; however, they compared the effectiveness between intramuscular injection and vaginal preparations, not between several vaginal preparations.

All four of the preparations are the same natural P4 agents, but their daily doses range from 90 mg to 300 mg, 600 mg, and 800 mg. Regardless of this, the fact that all four have the same pregnancy rates and MRs is extremely interesting. Over the history of vaginal preparations, the form of the preparations has undergone development from vaginal suppositories to gelatin capsules, bioadhesive gel, and most recently, foam agents.^33^, ^34^, ^35^, ^36^, ^37^, ^38^, ^39^, ^40^, ^41^ Crinone and Lutinus, which are newer preparations, contain smaller amounts of P4, presumably because they have superior solubility and absorption rates. In addition, Crinone and Lutinus are applied with the use of an applicator. It has been reported that the use of an applicator to place the preparation deeper into the vagina allows it to be efficiently transferred to the endometrium.^42^ This is the likely reason why these two preparations require smaller amounts of P4.

The methods of application and doses currently recommended by the manufacturers of all four preparations are indicated for use in Fresh‐ET procedures.^43^, ^44^, ^45^, ^46^ As a result, it is unknown whether the same doses are effective when the preparations are used for HRT‐FET. It has been reported that higher doses are better when used in HRT‐FET. One such study reported that the pregnancy rate was higher when the dose of Crinone is double the recommended dose of 90 mg/d (ie, 180 mg/d).^47^ Since this was a retrospective study, bias may have been involved. A second such study reported that the pregnancy rate was higher when Utrogestan was used at a dose of 1200 mg/d rather than 900 mg/d.^48^ Because additional doses are required when the blood P4 level is <9 ng/mL on day 5 of the luteal phase, this study was not properly conducted. The third such study investigated three groups: 50 mg daily intramuscular injection of P4, Lutinus (200 mg twice daily), and Lutinus (200 mg twice daily) with a 50‐mg intramuscular injection of P4 every third day. They found that the vaginal preparation alone, that is, Lutinus (200 mg twice daily) had a worse outcome than the other two, and therefore, it should be avoided.^49^ However, the dose of Lutinus that was used differed from that recommended by the manufacturer.

The present study's results of LPS for HRT‐FET indicated that there were no significant differences between the four vaginal P4 suppositories in terms of CPR, FHR, or MR. Nevertheless, this study has some limitations. First, as it was a single‐center study, there may have been a patient bias, and as all patients were Japanese individuals, their BMI may have been lower than in other races; thus, they may have required smaller doses than women from other countries. Second, the drug amount presented by each pharmaceutical company is the recommended amount based on Fresh‐ET; hence, it is unknown whether the amount is enough in HRT‐FET.

Assisted reproductive treatments performed as LPS requires an extremely long time, which places a high degree of stress on the patient. Currently, the use of Frozen‐ET is increasing worldwide, so establishment of effective LPS for HRT‐FET is urgently needed. Future large‐scale multi‐center cohort studies on this issue are required in order to obtain improved results.

In conclusion, there was no clinically significant difference in pregnancy outcomes between Lutinus, Utrogestan, Luteum, and Crinone when used in HRT‐FET for LPS. The four vaginal preparations differ in the number of administrations and use of an applicator. The period of corpus luteum supplementation in ART is long and stressful for women. If there is no difference in the pregnancy rate between the four drugs, then women can choose their preferred vaginal P4.

## CONFLICT OF INTEREST

None.

## DISCLOSURES

*Human rights statement and informed consent*: This study was approved by the Ethical Committee of Kinutani Women's Clinic (ethical review number 2016‐1110‐1) and registered in the UMIN Clinical Trials Registry (name of the trial register: exploratory test to investigate the CPR between four vaginal progesterones: Lutinus vaginal tablet (300 mg/d), Utrogestan vaginal capsule (600 mg/d), Luteum vaginal suppository (800 mg/d), and Crinone vaginal gel (90 mg/d), registration number: UMIN000032997). All procedures followed were in accordance with the ethical standards of the responsible committee on human experimentation (institutional and national) and with the Helsinki Declaration of 1964 and its later amendments. Informed consent was obtained from all patients included in the study. *Animal studies*: This article does not contain any study with animal participants that have been performed by any of the authors.
